# The jojoba genome reveals wide divergence of the sex chromosomes in a dioecious plant

**DOI:** 10.1111/tpj.15509

**Published:** 2021-10-08

**Authors:** Othman Al‐Dossary, Bader Alsubaie, Ardashir Kharabian‐Masouleh, Ibrahim Al‐Mssallem, Agnelo Furtado, Robert J. Henry

**Affiliations:** ^1^ Queensland Alliance for Agriculture and Food Innovation University of Queensland Brisbane 4072 Australia; ^2^ College of Agriculture and Food Sciences King Faisal University Al Hofuf 36362 Saudi Arabia; ^3^ ARC Centre of Excellence for Plant Success in Nature and Agriculture University of Queensland Brisbane 4072 Australia

**Keywords:** sex chromosomes, sexual dimorphism, dioecious plants, jojoba, Simmondsia chinensis

## Abstract

Most flowering plants are hermaphrodites, but around 6% of species are dioecious, having separate male and female plants. Sex chromosomes and some sex‐specific genes have been reported in plants, but the genome sequences have not been compared. We now report the genome sequence of male and female jojoba (*Simmondsia chinensis*) plants, revealing a very large difference in the sex chromosomes. The male genome assembly was 832 Mb and the female 822 Mb. This was explained by the large size differences in the Y chromosome (37.6 Mb) compared with the X chromosome (26.9 Mb). Relative to the X chromosome, the Y chromosome had two large insertions each of more than 5 Mb containing more than 400 genes. Many of the genes in the chromosome‐specific regions were novel. These male‐specific regions included many flowering‐related and stress response genes. Smaller insertions found only in the X chromosome totalled 877 kb. The wide divergence of the sex chromosomes suggests a long period of adaptation to diverging sex‐specific roles. Male and female plants may have evolved to accommodate factors such as differing reproductive resource allocation requirements under the stress of the desert environment in which the plants are found. The sex‐determining regions accumulate genes beneficial to each sex. This has required the evolution of many more novel sex‐specific genes than has been reported for other organisms. This suggest that dioecious plants provide a novel source of genes for manipulation of reproductive performance and environmental adaptation in crops.

## Significance Statement

This is the first report comparing the whole genome sequences of male and female plants of any species. This manuscript reports the discovery of a large genome‐level divergence between male and female plants based upon the first whole genome sequencing analysis of male and female dioecious plants.

## INTRODUCTION

Sex separation found in dioecious plants has evolved in a small portion of plant species for which this must offer some selective advantage (Leite Montalvão *et al*., 2020). Many different plant families have dioecious species, found in diverse environments. Male and female dioecious plants may differ in their needs for environmental resources due to their distinctive reproductive roles (Thomas and LaFrankie, 1993). Natural selection for reproductive success has played a crucial role in sexual differentiation (Kumar et al., 2016).

Sex differentiation and sex‐specific chromosomes have been extensively studied due to their significant role in plant evolutionary biology. The genetic basis of sexual differentiation in dioecious species has been found to be linked to nuclear genes on sex chromosomes (Baránková et al., 2020). Sex chromosomes in dioecious plant are often indistinguishable, and only a few of them show clear variation (Baránková et al., 2020). Many sex chromosomes have been reported in plants (Charlesworth, 2016; Smith and Smith, 1947) (Charlesworth, 2016; Smith and Smith, 1947) but have not been characterised in the same way as they have in animals (Negrutiu et al., 2001). In plant species, sex chromosome models broadly differ at the genus or species level (Charlesworth, 2013). A recent report found two sex‐specific genes on a sex chromosome in poplar (*Populus trichocarpa*) (Xue et al., 2020) and many other differences have been reported (Almeida et al., 2020; Atanassov et al., 2001; Massonnet et al., 2020) at the genome level. However, the genome sequences of male and female plants have not yet been reported.

There are many plants with homomorphic sex chromosomes with a small non‐recombining sex‐determining region for which the sequence has been reported (Charlesworth, 2016). However, here we report the chromosome‐level assembly of heteromorphic sex chromosomes in a plant.

Jojoba (*Simmondsia chinensis*) is a dioecious desert shrub that inhabits the Sonoran Desert between the United States and Mexico. This perennial woody plant is the only species in the family Simmondsiaceae (Kumar et al., 2019). Jojoba is used (Alotaibi et al., 2020; Arya and Khan, 2016; Kumar et al., 2019; Sturtevant et al., 2020) to produce a unique liquid wax easter (jojoba oil), which represents 45–55% of the jojoba female seed weight (Inoti, 2018). Jojoba oil has many important characteristics, which makes it a renewable candidate source of biodiesel production (Sandouqa and Al‐Hamamre, 2019). Beside the significant interest in the oil, its cultivation has expanded in arid to semi‐arid regions because of its ability to tolerate different stresses and poor soil environments (Wang et al., 2019). The morphological and physiological characteristics of jojoba play an essential role in its adaptation to various stresses as well as its sex‐based variation. In a study of biochemical and physiological responses to drought, male jojoba plants expressed higher tolerance than females (Kumar et al., 2016), suggesting very different environmental responses to stress. Jojoba seed germination is male‐biased, with a successful male:female germination ratio of 5:1 having been reported (Heikrujam et al., 2015a). This creates a challenge in production with plant sex not being revealed until a very late growing stage. Several efforts have been made to identify the genetic basis of sexual dimorphism using previously identified sex‐related genetic markers in other plants (Heikrujam et al., 2015b; Hosseini et al., 2011). However, to understand the sex differences reference genomes for both sexes are needed. The expansion of genomic resources for jojoba will create an opportunity to address differences between the sexes and sex chromosome evolution in a dioecious plant.

Recently, the first reference genome of a jojoba plant of undefined sex was reported (Sturtevant et al., 2020). The 887‐Mb genome of jojoba was comprised of 26 chromosomes including 23 490 protein‐coding genes and suggested an ancient whole genome triplication with no recent duplications in the jojoba genome. The study focused on machinery for lipid synthesis and storage based on transcriptome, proteome, and lipidome data.

However, to define the sexual dimorphism in jojoba, separate reference genomes for both sexes are required. We now report the sequencing of male and female jojoba plants and analysis of the genomes to define in more detail the differences between the genome sequences of a male and female plant for the first time.

## RESULTS AND DISCUSSION

### Structural differences between male and female jojoba genomes

**Figure 1:**
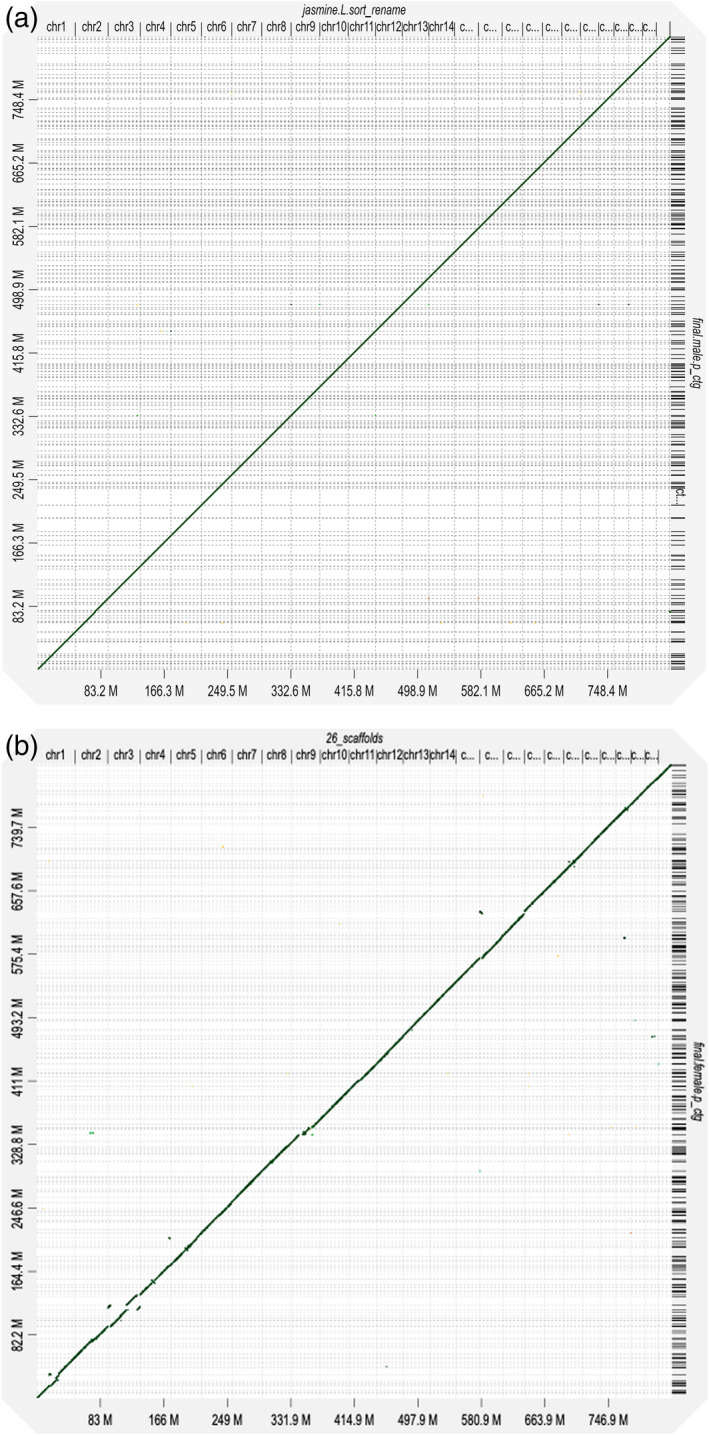
Comparison of (a) HiFi male assembly and (b) HiFi female assembly against male genome chromosome‐level assembly (Omni‐C scaffolding). The *x*‐axis shows the 26 pseudomolecules representing the chromosomes in the male genome and the *y*‐axis shows the contigs of the HiFi assembly of the (a) male and (b) female genome.

The genomes of male and female jojoba plants were sequenced using long‐read sequencing (Murigneux et al., 2020) (PacBio HiFi) generating genome assemblies produced using Improved Phased Assembly (IPA) (Sharma et al., 2021). The male assembly was significantly larger than the female assembly (male 832 Mb [N50 5.7 Mb and Benchmarking Universal Single‐Copy Orthologs [BUSCO] completeness 96.9%] and female 822 Mb [N50 4.9 Mb and BUSCO completeness 97.4%]). The k‐mer analysis, while supporting a larger male genome, gave a lower estimate of genome size than that provided by the HiFi assembly (Table S1), presumably due to the nature of short sequence reads and the challenge of estimating genome size for genomes with highly repetitive elements (Guo et al., 2015; Panfilio et al., 2019). The male genome had 1.53% heterozygosity and the female 1.31% (Figure S1). Proximity ligation of chromatin fragmented by a sequence‐independent endonuclease (Omni‐C) was used to produce a chromosome‐level assembly of the male jojoba genome (Table S2). Assembly of the HiFi contigs produced pseudomolecules representing all 26 chromosomes (Table S3, Figure 1). Completeness of this assembly was 97% based upon analysis of the presence of conserved viridiplantae genes (BUSCO, Table S4). Jojoba has 52 chromosomes and some have suggested the possibility of it being a tetraploid (*n* = 13, 4*n* = 52) (Tobe et al., 1992). However, the assembly and lack of evidence for homology between the pseudomolecules indicated that jojoba should be considered as a diploid (*n* = 26). Analysis of the syntenic regions in the genome identified 346 putative homologous genes and Ks analysis showed evidence of two whole genome duplication events: an ancient whole genome duplication and a more recent one (Figure S2). The jojoba genome has apparently gone through a diploidization process, as suggested by cytological evidence that the chromosomes behaved like those of a diploid (Tobe et al., 1992). Links between polyploidy and the evolution of dioecy have been suggested but remain unclear (Ashman et al., 2013).

**Figure 2:**
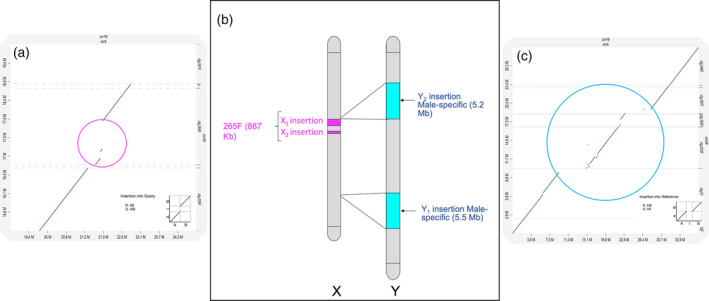
Illustration of sex chromosomes in jojoba. The numbers indicate the name and position of contigs. (a) 2D plot of alignment between the female‐specific region within contig 265F and the Y chromosome assembly. The purple circle shows the two insertions in contig 265F. (b) The X chromosome includes 877 Kb in two insertions which are not found in the Y chromosome or other parts of the male genome (female‐specific). The Y chromosome contains two male‐specific insertions, which are not found in the X chromosome or elsewhere in the female genome. The total length of the male‐specific insertions is approximately 10.7 Mb (5.5 Y_1_ + 5.2 Mb Y_2_). (c) 2D dot plot of alignment between the female genome assembly (contigs) and the Y chromosome assembly. The blue circle shows the two insertions into the Y chromosome (ChrY9).

The male and female genomes were compared by aligning the HiFi contigs produced by assembly of the PacBio reads with the HI‐C derived pseudomolecules (Figure 2). The results showed that chromosome 9 (chromosomes were numbered in order of decreasing size) differs between the male and female and corresponds to a sex chromosome. This comparative genomic analysis showed a significant difference in the length of chromosome 9 between male and female plants. This suggests an XY chromosome system, in which the Y chromosome (male‐specific) is much longer than the X chromosome. The 2D dot plot of the assembled male contigs against the chromosome‐level assembly showed that the total length of the Y chromosome was 37.6 Mb, while the X chromosome was only 26.9 Mb, that is, 10.7 Mb shorter than the Y chromosome (Figure 2). This was largely due to the presence of two large insertions in the Y chromosome, one 5.5 Mb (insertion Y_1_) and the other 5.2 Mb (insertion Y_2_) (Figure 2). These two insertions are genome regions found only in the male (male‐specific). We also found insertions on the X chromosome within contig 265F (ctg.265f) that were not present in chromosome Y (Figure 2). The total length of these insertions was 887 kb, including a 713‐kb insertions (insertion X_1_) and a 174‐kb insertion (insertion X_2_). These specific insertions in the X and Y chromosomes together explain the genome size difference between the male and female, which was first detected in the long‐read (IPA) assembly (Figure 1) and in the k‐mer analysis of short reads (Figure S1). This is the first genomic sequencing of an XY chromosome system in dioecious plants and suggests that these two large insertions (in the Y chromosome) may largely explain the wide divergence between the two sexes. This differs from the widely reported mammalian system, in which the Y chromosome is much shorter than the X chromosome. Mammalian sex chromosomes are usually thought to be derived from autosomes that have gone through many evolutionary processes, especially in speciation (Presgraves, 2008). However, the larger Y chromosome in jojoba contrasts with the classical view in animals that the Y chromosomes represent a highly degenerate version of the X chromosome, in which the Y chromosome has lost most of the ancestral genes (Bachtrog, 2013) and is supported by other reports of large X chromosomes in some plants (Charlesworth, 2016). We suggest that the Y and X insertions may have evolved by segmental genome duplication and divergence events during the evolution of dioecy in this species.

A total of 426,380 transcript isoforms were produced from 296.6 Mb of PacBio long‐read cDNA data. These transcripts were mapped to both the male and female assemblies, 99.8% and 99.6% mapping successfully for male and female, respectively. The BUSCO and RNA alignment results indicated a high‐quality genome assembly with excellent completeness and accuracy for both sexes.

### Annotation of male and female genomes

Annotation of the whole genome shed more light on the characteristics of the sex chromosomes and the two insertions in the Y chromosome (Table 1). The whole genome annotation showed 1616 genes in the Y chromosome.

**Table 1:** Annotation of chromosome‐level assembly of male genomic sequences

Component	Gene#	Repeat elements (%)	LTRs (%)
Whole genome	35 089*	70.6	32.4
Chromosome Y	1616	62.9	28.2
Y_1_ insertion	248	59.7	40.3
Y_2_ insertion	181	48.0	30.6

LTR, long terminal repeat.

* 31 848 mRNAs, 424 tRNAs, 34 625 proteins, and 35 089 genes.

A large number of genes were found in the regions of the two insertions in the Y chromosome. Of the 429 genes in the two insertions in the Y chromosome, 248 and 181 genes belong to the Y_1_ and Y_2_ regions, respectively. Comparison of the genes in the male‐specific inserts in the Y chromosome showed that only three genes (with no match) in the Y_1_ region and none of the genes in the Y_2_ region had 90% similarity, establishing that all of the genes in these regions were distinct and not duplicates. Only one gene (with no match) was common between the Y_1_ and Y_2_ regions. This suggests that the male‐specific regions of the jojoba genome have more than 400 unique genes. Only one gene in these regions was also present on the other parts of the Y chromosome.

The identification of many sex‐specific genes in jojoba will aid the development of tests to distinguish male and female plants for deployment in commercial crops (Waser, 1984).

Analysis of many of the genes in the Y_1_ and Y_2_ insertions in the whole genome annotation resulted in matches with known genes. Of a total of 429 genes, 147 (87 from Y_1_ and 60 from Y_2_) had no matches, 14 matched hypothetical proteins, and 26 matched genes for uncharacterised proteins. This clearly indicates that many of the genes are poorly known and around one‐third of these sequences were unique with homologues not found in other organisms despite the strong evidence from their expression in RNA sequencing (RNA‐Seq) data (see details below), confirming they were not artifacts of annotation.

Many genes found in the Y_1_ and Y_2_ regions were associated with flowering, which might be expected to vary between male and female (Table 2). These included embryo sac development arrest (Pagnussat et al., 2005) and FTIP1 (Liu et al., 2012) in YI and *HOTHEAD* (Kurdyukov et al., 2006), stamen‐specific protein (Nacken et al., 1991), protein gamete expressed 2 (Mori et al., 2014), self‐incompatibility S1 domain‐containing protein (Williams et al., 2015), and F‐box protein genes (Xu et al., 2009) in Y_2_.

**Table 2:** Examples of notable male‐specific genes associated with flowering or floral development and markers found in the Y_1_ and Y_2_ chromosome regions

Chromosome region	Gene or marker name	Reference
Y_1_	*Embryo sac development arrest*	Pagnussat *et al*. (2005)
	*FTIP1*	Liu *et al*. (2012)
	Jojoba male‐specific marker ISSR‐UBC‐807	Sharma *et al*. (2008)
	Jojoba male‐specific marker ISSR‐VIS‐11	Heikrujam *et al*. (2014a)
	Jojoba clone pkmssj male‐specific	Heikrujam *et al*. (2014b)
Y_2_		
	*HOTHEAD*	Kurdyukov *et al*. (2006)
	*Stamen specific protein*	Nacken *et al*. (1991)
	*Protein gamete expressed 2*	Mori *et al*. (2014)
	*Self‐incompatibility S1 domain containing protein*	Williams *et al*. (2015)
	*F‐box protein genes*	Xu *et al*. (2009)
	*APETALA2*	Okamuro *et al*. (1993)
	*MADS‐box transcription factor*	Teo *et al*. (2019)
	*Perpetual Flowering 2 (PEP2*)	Lazaro *et al*. (2018)

Abbreviations: ISSR, inter‐simple sequence repeat.

More focused annotations focused on just annotating the Y_1_ and Y_2_ regions rather than the whole genome revealed additional genes related to flowering or floral development. A gene with homology to a gene regulating floral development, *APETALA2* (Okamuro et al., 1993), was found in Y_2_. *Perpetual Flowering 2* (*PEP2*) (Lazaro et al., 2018) and a MADS‐box transcription factor (Teo et al., 2019), both of which have been linked to control of flowering, were found in this region. MADS‐box genes have been linked to control of the transition to flowering (Teo et al., 2019). Genes controlling flower development are to be expected on sex chromosomes of dioecious plants. A *Pollenless 3* gene (Sanders et al., 1999) was found to be located on the distal ends of both the Y and X chromosomes of jojoba. Male‐specific expression of genes on the X chromosome has been found in humans (Lercher et al., 2003) and plants may also have genes with sex‐specific expression on both or either of the X and Y chromosomes. All three male‐specific inter‐simple sequence repeat markers (Heikrujam et al., 2014a,b; Sharma et al., 2008) that have been reported for jojoba were also found to be specifically located in the Y_1_ region. The sex‐determining genes reported for poplar (Mueller et al., 2020) and date palm (*Phoenix dactylifera*; Torres et al., 2018) were found elsewhere in the jojoba genome ‐and appear to not have a role in sex determination in jojoba. Independent evolution of dioecious plants has not followed the same path.

The mapping of RNA‐Seq reads to genes in these two inserts confirmed that they were expressed, with more than 94% of genes having 30% or more of their length covered by transcribed sequences. Long terminal repeat (LTR) elements were more abundant in the male‐specific insertions on the Y chromosome (Table 1). The presence of repetitive elements is a characteristic of plant sex chromosomes (Hobza et al., 2017).

**Figure 3:**
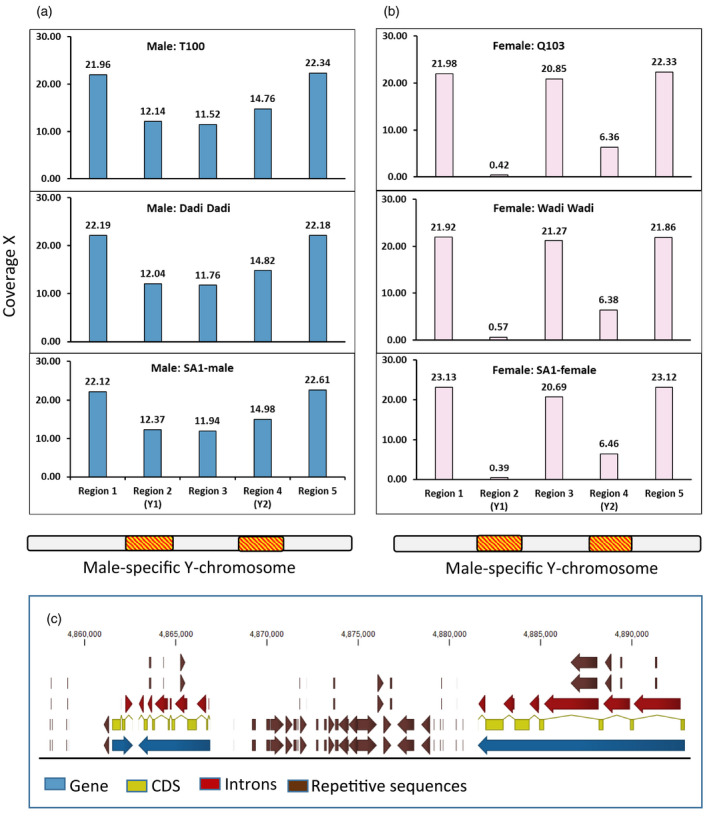
Characterisation of male‐specific regions in the Y chromosomes. Illumina paired‐end read mapping coverage along the male‐specific Y chromosome (Figure 2) as the reference. Region 1 (distal end); region 2 (male‐specific Y_1_); region 3 (region between Y_1_ and Y_2_); region 4 (male‐specific Y_2_); and region 5 (distal end). (a) SA1‐Male (Saudi), Dadi Dadi, and T100: jojoba male genotypes. (b) SA1‐Female (Saudi), Wadi Wadi, and Q103: Jojoba female genotypes. Region‐wise average coverage values are normalised with respect to the total nucleotides of paired‐end reads of the SA1‐Female (Saudi) genotype mapped to the jojoba male‐specific Y chromosome. (c) Repetitive element mapping track of a region of the jojoba male‐specific Y_1_ chromosome region. The sex‐specific regions are gene‐rich. The space between the coding sequences (intergenic spaces and introns) is almost completely filled with repetitive sequences.

To establish that these differences were consistently found in the genomes of male and female jojoba plants, analysis of the sex‐specific regions of several male and female plants was achieved by mapping Illumina reads from three male and three female genotypes to the Y chromosome assembly (Figure 3). Illumina reads from female genotypes mapped only to the common regions and not to any significant extent to the Y_1_ and Y_2_ insertions. Illumina reads from the male genotypes mapped across the whole chromosome with higher coverage in the common regions due to contribution of reads from both the X and Y chromosomes and lower coverage in the inserts contributed by the Y chromosome. The distal parts of the chromosomes showed mapping of both X and Y chromosome reads with 21.9–23.1× coverage in all six genotypes. The male‐specific insertion Y_1_ (as marked on Figure 2) had 12.1–12.4× coverage for the three male genotypes and 0.4–0.6× coverage for the three female genotypes, demonstrating the presence of a single copy of these chromosome regions in the males only. The second male‐specific region (Y_2_ in Figure 2) had a slightly higher mapping from the male, suggesting some reads with homology in the female, and this was confirmed by the 6× coverage with female reads. The region between the two insertions (Y_1_ and Y_2_) was found to be more complex. This region had insertions specifically on the X chromosome (Figure 2) that did not show here with a male reference chromosome. Some smaller male‐specific insertions were also present (as evident in Figure 2) resulting in an average coverage over the whole region of 11.5–11.9× for the males and 20.7–21.3× for the females. All three male and all three female genotypes had a very similar mapping coverage, confirming the sex rather than genotype specificity of these structures. The overlay of repeat element data and coding regions showed a perfect packaging of repetitive elements and LTRs in intergenic regions and introns of the genes (Figure 3). This indicates that transposons and LTRs have been integrated into the landscape of the genes to create a high content of both genes and repetitive elements. The genome had a high repetitive sequence content being 70% in the male genome (Table S5). The proportions of the male‐specific regions that were composed of repetitive elements were 59.7 and 48.0% for the Y_1_ and Y_2_ insertions, respectively. LTR Gypsy elements were more common in the male‐specific inserts and were reported as sex‐specific in poplar (Xue et al., 2020).

Phylogenetic analysis has placed jojoba in the Caryophyllales, and this was confirmed by analysis of homology of jojoba gene sequences with those of other species. The most closely related sequences, based on a functional annotation analysis in OmicsBox v2.0 for the isoseq transcripts (Table S6) and coding sequences from the HiFi assembly (Table S7), were from beetroot (*Beta vulgaris* subsp. *vulgaris*), quinoa (*Chenopodium quinoa*), and spinach (*Spinacia oleracea*).

### Evolution of male and female genomes

Dioecy has evolved many times in plants and is found in around 7% of genera and 6% of species of flowering plants (Renner and Ricklefs, 1995). Substantial morphological differences have been found between male and female jojoba plants, especially when grown under dry conditions (Inoti et al., 2015; Kohorn, 1994). These differences can now be associated with the substantial number of additional genes located on the Y chromosome in male plants. Many different explanations of the causes of the evolution of dioecious plants have been explored (Thomson and Brunet, 1990). Divergent evolution of males and females may be driven by selection for reproductive success in the desert environment (Thomson, 2006). Male seedlings are more tolerant of drought stress and their more rapid growth and development may allow them to reach flowering early. This may be due to stress response genes found in the male‐specific insertions on the Y chromosome. For example, a gene encoding a protein dehydration response protein (Choudhary et al., 2009; Vessal et al., 2020) was located in Y_1_. Female plants have been shown to devote significant proportions of their resources to seed production (Kohorn, 1994). Success may be enhanced by greater root growth, allowing the female plants to establish for the longer growth phase required to support seed production. The ratio of male to female plants in wild populations has only a slight bias towards more males (Waser, 1984), while in cultivation males may predominate when grown under stress (Inoti et al., 2015). Many studies on jojoba cultivation have noted this high male to female ratio (Agrawal et al., 2007). Hosseini et al. (2011) also cited Agrawal et al. (2007), indicating this abnormal ratio. So far, there is no evidence explaining how this ratio could be controlled genetically. The presence of an XY chromosome system suggests equal numbers of male and female seeds should be produced. However, Cole (1979) studied the effect of environmental factors on aberrant sex ratios in jojoba. They established a 3‐month study and planted seeds to create 14 populations on north‐ and south‐facing slopes in Arizona, US. They reported 51% of female jojoba on north‐facing slopes but only 45% on south‐facing (hotter and dryer) slopes. They suggested that there is some correlation between orientation of the slope and the male:female ratio, although many other environmental factors such as water stress and high temperature can increase female mortality and change the sex ratio in the population. The much higher abundance of males reported in cultivation may be due to higher survival rates of male seedlings that have a greater stress tolerance. In the wild, females that survive the seedling stage may survive longer with a better root system, balancing the greater early survival of the males as seedlings. The large number of male‐specific genes in the male‐specific parts of the Y chromosome may account for the very significant sexual dimorphism of morphological and growth traits in this species. This divergence of the sexes allowing adaptation to their sex‐specific roles in reproduction may be the critical selective advantage that has led to the evolution of dioecious plants. The identification of large numbers of sex‐specific genes in jojoba provides an opportunity to explore the functions that have diverged with sex separation.

Climate change may adversely impact on the sex ratio (Hultine et al., 2016) in wild populations of jojoba but the direction of any change is not clear. Dioecious plants may be at greater risk of extinction if climate change results in a rapid development of sex imbalance in their populations (Tognetti, 2012). Analysis of the sex‐specific genes discovered in this study and determination of their functions may help determine the nature of the risk faced by being dioecious.

Dioecious plants have arisen many times independently in divergent lineages. This divergence may have followed similar (or parallel) paths in different species but may be different in differing environments posing divergent selection pressures. The extent of divergence discovered here is much greater than that reported in other plants or in animals. For example, the presence of 1616 genes on the jojoba Y chromosome with 429 genes found in Y chromosome‐specific insertions can be compared with only 78 protein‐coding genes on the human Y chromosome (Bachtrog, 2013). Differences in some species seem relatively small (Martine et al., 2016). The substantial divergence in size of the sex chromosome in jojoba contrasts with the view that these chromosomes are often not easily distinguished in the cytology of dioecious plants (Zhou et al., 2020). The extreme stress imposed by the desert environment may drive strong selection for diversification to achieve reproductive success for males and females. Many other dioecious desert plants have been the subject of genetic research (Case and Barrett, 2004; Wolfe and Shmida, 1997). The extent to which other desert plants have undergone parallel evolution and substantial sex divergence in this extreme environment will be revealed as more dioecious desert plants and dioecious plants in general are subjected to genome analysis (Charlesworth, 2013). The common presence of repeat elements, specifically LTRs, in sex chromosomes has been explained by their potential to block recombination and as a result drive chromosome divergence (Charlesworth, 2017). The presence of common or functionally similar sex‐specific genes may also be found as we explore the genomes of dioecious plants and gene interactions in sex chromosomes (Harkess et al., 2020).

The analysis of the genomes of dioecious plants can be expected to reveal a great diversity of options for crop improvement, providing a rich source of novel genes for both the manipulation of reproduction and environmental adaptation in crops.

## EXPERIMENTAL PROCEDURES

### Plant materials

Jojoba seed were obtained from King Faisal University (KFU) Al‐Hofuf, Saudi Arabia (25°16'15.1"N, 49°42'42.6"E) during July 2019. Two jojoba plants, male and female, were identified and assigned for leaf tissue collection. For each sex, 10 g of healthy plant leaf tissue was collected and manually ground under liquid N_2_ using motor and pestle. The ground tissue was immediately suspended in a 50‐ml Falcon tube containing 40 ml of 2% cetyl trimethyl‐ammonium bromide (CTAB) extraction buffer.

The leaf tissues from two different varieties per sex (Daddi‐Daddi and T100 for male; Wadi‐Wadi and Q103 for female) were collected randomly from the ‘Chris‐Egan’ farm at Inglewood (151°4'.20"E, 28°25'13"S), Queensland, Australia. The collected leaf tissues were snap‐frozen in liquid nitrogen and placed in dry ice followed by preservation at −80°C. The leaf tissues were ground to a fine powder using a Tissue Lyser‐II (Qiagen, Valencia, CA, USA) at a frequency of 30 Hz for 30 sec prior to DNA extraction.

### Genomic DNA isolation and sequencing

Genomic DNA from mature leaf tissue of jojoba male and female plants from Saudi Arabian and Australian varieties were extracted following a modified CTAB method that was published previously (Carroll et al., 1995; Furtado, 2014). The modification took place in several steps through the extraction protocol. In the step of adding nuclear lysis buffer and 5% Sarkosyl solution, 0.06 g of sodium sulphite was also added to the extraction mix. The mixture was incubated at 50°C for 45 min with repeated inversions (5–8 times) and was followed by adding 10 ml of chloroform. During the DNA washing steps, 5 ml of 70% ethanol was added. The extracted DNA was quantitatively and qualitatively evaluated using agarose gel electrophoresis (0.7%, 110 V for 45 min) and a NanoDrop 8000 Spectrophotometer (Thermo Scientific, Waltham, MA, USA), respectively. The DNA samples from both sexes (male and female) were analysed using two different sequencing platforms: PacBio (Menlo Park, CA, USA) and Illumina (San Diego, CA, USA). The sequencing libraries for short (NextSeq2000) and long reads required 0.025 and 20 µg of DNA, respectively. The sequencing libraries were generated based on the manufacturer’s protocols. Illumina sequencing yielded the following amounts of sequence data: Saudi male 71.5 Gb, Saudi female 72.3 Gb, Daddi 34.3 Gb, T100 33.7 Gb, Wadi 38.8 Gb, Q103 34.5 Gb.

An Omni‐C library was prepared by extracting chromatin after fixation in the nucleus using formaldehyde. The extracted chromatin ends were digested using DNAse I and then repaired and re‐ligated to a biotinylated bridge adapter followed by proximity ligation of adapter‐containing ends. Furthermore, the crosslinks were reversed, and the DNA was purified. A sequencing library was prepared using NEBNext Ultra enzymes and Illumina‐compatible adapters. The library was sequenced using an Illumina HiSeqX platform.

### Genome size estimation

K‐mer frequency distribution analysis was used to estimate jojoba male and female genome sizes and heterozygosity rates using KAT software v2.4.2 (Mapleson et al., 2017). K‐mer frequency distribution was calculated and plotted using 71.5 and 72.3 Gb of Illumina short‐read sequencing data for male and female to determine the total number of k‐mers of length 27 by means of the count function in Jellyfish (Marçais and Kingsford, 2011). The length of k‐mers (27‐mers) was chosen based on jojoba genome characteristics. Moreover, the k‐mer frequency peak of the reads (M) corresponded to the sequencing depth (N), read length (L), and k‐mer length (K) and by the following formula: M = N × (L − K + 1)/L (Dong et al., 2010). The peak of the 27‐mer frequency from the paired‐end reads of jojoba was 63 and 65 for male and female, respectively (Figure S1). The genome sizes were estimated to be 724.9 and 727.4 Mb for male and female, respectively.

### Total RNA isolation and sequencing

A mixture of total RNA was obtained from a pilot water stress experiment. The water stress experiment was designed to have 10 time‐points and two replications, and one plant was designated to every replicate. The 10 time‐points were as follows: day 0, day 4, day 7, day 9, day 11, day 14, day 16, day 18, day 22, and day 25. Twenty 3‐month‐old jojoba plants were used in this experiment. The experiment involved withholding water from initially well‐watered plants for 25 days. Leaf tissue collection took place at every time‐point, and 20 random leaves were collected into micro‐perforated polyethylene (PET) bags from every replication and snap‐frozen in liquid nitrogen. The collected leaves from every sample were ground to a fine powder using a Tissue Lyser‐II (Qiagen) or a Mixer Mill‐400 (Retsch, Haan, Germany) at a frequency of 30 Hz for 30 sec. The fine powder of each sample was transferred into 50‐ml Falcon tubes and stored at −80°C.

A two‐step protocol, including a cetyltrimethylammonium bromide (CTAB) method followed by a Qiagen RNeasy Plant mini kit (#74134, Qiagen), was used to ensure the complete removal of contaminating genomic DNA (Yang et al., 2008). The qualitative and quantitative evaluation of the total RNA extracts were accomplished using a NanoDrop 8000 Spectrophotometer (ThermoFisher Scientific, Wilmington, DE, USA) and a 2100 Agilent Bioanalyzer (Agilent Technologies, Santa Clara, CA, USA), respectively. Samples from day 0 to day 14 were mixed to use in jojoba genome annotation. RNA integrity number (RIN) for the mixed RNA samples ranged from 2.20 (day 14) to 5.30 (day 0) due to possible contaminants. The mixed RNA sample was sequenced using an Illumina platform.

### Genome assembly

The final *de novo* assembly of PacBio reads was conducted using IPA v:1.3.1 with default parameters. Assembly statistics were calculated using the Quality Assessment Tool (QUAST) v5.1.0 based on a contig size of ≥500 bp (Gurevich et al., 2013). The completeness of the reference genome assembly was assessed using BUSCO v4.1.2 with the viridiplantae_odb10 database (Gurevich et al., 2018).

### Assembly scaffolding using Hi‐Rise

The jojoba male *de novo* assembly and Omni‐C library reads were inputted into HiRise to scaffold the genome assemblies using proximity ligation data (Putnam et al., 2016). Sequences from the Dovetail Omni‐C library were mapped to the draft assembly using a modified SNAP read aligner (http://snap.cs.berkeley.edu). The separations of Dovetail Omni‐C read pairs aligned within the draft assembly were examined by HiRise to create a likelihood model for genomic distance between read pairs. The generated model was employed to determine and separate putative mis‐joins, to score prospective joins, and to make joins above a threshold.

### Transcriptome sequencing

Equal volumes of five RNA samples representing three time‐points (day 0, day 8, and day 14) of a water stress experiment were mixed and sequenced using PacBio long‐read cDNA sequences. The cDNA was amplified and filtered following the Iso‐Seq protocol into standard and long transcript libraries. The two libraries were quantified using two SMRT cells of the Pacific Bioscience (PacBio) Sequel II platform, generating 296.6 Mb of data (426 380 isoforms).

### Assessment of genome assembly

Jojoba genome assembly completeness was assessed using BUSCO and a jojoba transcriptomic database. The jojoba genome assembly was searched against 425 conserved single‐copy orthologs in the viridiplantae_odb10 BUSCO v4.1.2 dataset with default parameters. Furthermore, transcript isoforms that were produced by PacBio long‐read sequencing of cDNA were used to confirm the high quality of the genome assembly. The isoforms were aligned to the final assembly using CLC Genomics Workbench v20.0.4 (CLC‐GWB, CLC Bio‐ Qiagen, Aarhus, Denmark) with default parameters. High‐molecular‐weight genomic DNA from three male jojoba genotypes (Male‐SA1, Dadi Dadi, and T‐100) and from three female genotypes (Female‐SA1, Wadi Wadi, and Q103) were subjected to 150‐bp paired‐end read sequencing on the Illumina NovaSeq 6000 platform. The sequence reads were trimmed at 0.01 quality limit (equivalent to a Fred Score of 30 or above) and sequence read data between 22 and 33 million paired‐end reads from the male and female genotypes were used for mapping to the Saudi jojoba male assembly as the reference. Mapping parameters included: match score 1, mismatch cost 2, insertion/deletion cost 3, length and similarity fraction of 0.8 each, and ignoring non‐specific matches (CLC Genomics Workbench, Qiagen). Total nucleotides of the paired‐end reads mapped to the Saudi jojoba female assembly (as reference) were used to normalise the nucleotide coverage of specific regions (regions 1 to 4) for each of the jojoba genotypes.

### Genome annotation

Whole genome annotation was conducted by Dovetail Genomics. Coding sequences from *S. chinensis* (Sturtevant et al., 2020), *Theobroma cacao, Arabidopsis thaliana*, and *Oryza sativa* were used to train the initial *ab initio* model for *S. chinensis* using AUGUSTUS software (version 2.5.5). Six rounds of prediction optimisation were done with the software package provided by AUGUSTUS. The same coding sequences were also used to train a separate *ab initio* model for *S. chinensis* using SNAP (version 2006‐07‐28). RNA‐Seq reads were mapped onto the genome using the STAR aligner software (version 2.7) and intron hints were generated with the bam2hints tools within AUGUSTUS. MAKER, SNAP, and AUGUSTUS (with intron–exon boundary hints provided from RNA‐Seq data) were then used to predict genes in the repeat‐masked reference genome. To help guide the prediction process, Swiss‐Prot peptide sequences from the UniProt database were downloaded and used in conjunction with the protein sequences from *S. chinensis*, *T. cacao*, *A. thaliana*, and *O. sativa* to generate peptide evidence in the MAKER pipeline. Only genes that were predicted by both SNAP and AUGUSTUS software were retained in the final gene sets. To help assess the quality of the gene prediction, AED scores were generated for each of the predicted genes as part of the MAKER pipeline. Genes were further characterised for their putative function by performing a homology search of the peptide sequences against the UniProt database. tRNAs were predicted using the software tRNAscan‐SE (version 2.05).

An alternate male and female genome annotation and annotation of male‐specific parts of the genome was conducted using the HiFi assemblies for both sexes. The web‐based Genome Sequence Annotation Server (GeneSAS) was employed to annotate the draft genome assemblies (Humann et al., 2019). GeneSAS is a platform that combines multiple annotation tools for whole genome structural and functional annotations.

### Repeat annotation

Repeat families found in the genome assemblies of *S. chinensis* were identified *de novo* and classified using the software package RepeatModeler (version 2.0.1). RepeatModeler depends on the programmes RECON (version 1.08) and RepeatScout (version 1.0.6) for the *de novo* identification of repeats within the genome. The custom repeat library obtained from RepeatModeler were used to discover, identify, and mask the repeats in the assembly file using RepeatMasker (Version 4.1.0). The repetitive elements of the male and female jojoba genomes including tandem repeats and transposable elements were identified using RepeatModeler, which uses two *de novo* repeat finding programmes (Recon and RepeatScout) (Bao et al., 2015).

## CONFLICT OF INTEREST

The authors declare no conflict of interest.

## AUTHOR CONTRIBUTIONS

Study conception and design: all authors. Data collection: OA and BA (BA contributed most to the annotation and OA to the assembly). All authors contributed to analysis and interpretation of results and drafting the manuscript, reviewed the results, and approved the final version of the manuscript.

## Supporting information


**Table S1**. Jojoba male and female genome assemblies using Improved Phase Assembly (IPA) v:1.3.1.
**Table S2**. Quality Assessment Tool (QUAST) statistics of the jojoba (*Simmondsia chinensis*) male genome assembly using Dovetail HiRise v2.0.
**Table S3**. The lengths of 26 jojoba pseudochromosomes based on chromosome‐level Dovetail HiRise assembly.
**Table S4**. Benchmarking Universal Single‐Copy Orthologs (BUSCO) analysis of jojoba male results for both assemblers: HiRise and Improved Phase Assembly (IPA).
**Table S5**. The percentage of repeat family sequences in male and female jojoba genomes v1.0.11.
**Table S6**. The top four jojoba closely related species based on a functional annotation analysis in OmicsBox v2.0 for the jojoba isoseq reference. A close relationship was shown between jojoba and the following species: quinoa (*Chenopodium quinoa*), beetroot (*Beta vulgaris* subsp. *vulgaris*), spinach (*Spinacia oleracea*), and grape vine (*Vitis vinifera*).
**Table S7**. The top three jojoba closely related species based on a functional annotation analysis in OmicsBox v2.0 for coding sequences (CDSs) from the jojoba male HiFi assembly. A close relationship was shown between jojoba and the following species: beetroot (*Beta vulgaris* subsp. *vulgaris*), quinoa (*Chenopodium quinoa*), and spinach (*Spinacia oleracea*).
**Figure S1**. The k‐mer distribution and coverage of sequencing reads at K = 27 for (a) male and (b) female. Peaks with single and double asterisks were evaluated as k‐mer species derived from heterozygous (k‐mer frequency = 13) and homozygous (k‐mer frequency = 63) sequences for male and heterozygous (k‐mer frequency = 12) and homozygous (k‐mer frequency = 65) sequences for female.
**Figure S2**. Syntenic comparison of the jojoba genome. A dot plot showing the syntenic gene pairs (totally 346) in the jojoba genome. Each dot shows one synteny gene pair (left: colour‐coded based on Ks rates in Figure 2). The grey lines (on the *x* and *y*‐axes) represent chromosomes.
**Figure S3**. Ks analysis (synonymous distribution) of the jojoba genome. The median peak (orange) with a Ks value of 0.2 shows an early whole genome duplication (WGD) event (syntenic orthologs). The red peak with a Ks value of 0.5 shows the younger (second) WGD (syntenic out‐paralogs). The green and blue columns are noises.

## Data Availability

Jojoba reference genome sequence data (Illumina, PacBio, and Hi‐C interaction reads), the final genome assemblies, structural and functional annotations for both male and female, and the long‐read transcriptome are available at the NCBI website under Bioproject ID PRJNA694450. The data is also available the Genome Warehouse website under bioProject number PRJCA006974
