# Supplementary material for: The jojoba genome reveals wide divergence of the sex chromosomes in a dioecious plant
Source: Plant J. 2021 Oct 8;108(5):1283–94. doi: 10.1111/tpj.15509 (PMC9293028; doi:10.1111/tpj.15509)
Supplement: Supplementary file 1 — Table S1. Jojoba male and female genome assemblies using Improved Phase Assembly (IPA) v:1.3.1. Table S2. Quality Assessment Tool (QUAST) statistics of the jojoba (Simmondsia chinensis) male genome assembly using Dovetail HiRise v2.0. Table S3. The lengths of 26 jojoba pseudochromosomes based on chromosome‐level Dovetail HiRise assembly. Table S4. Benchmarking Universal Single‐Copy Orthologs (BUSCO) analysis of jojoba male results for both assemblers: HiRise and Improved Phase Assembly (IPA). Table S5. The percentage of repeat family sequences in male and female jojoba genomes v1.0.11. Table S6. The top four jojoba closely related species based on a functional annotation analysis in OmicsBox v2.0 for the jojoba isoseq reference. A close relationship was shown between jojoba and the following species: quinoa (Chenopodium quinoa), beetroot (Beta vulgaris subsp. vulgaris), spinach (Spinacia oleracea), and grape vine (Vitis vinifera). Table S7. The top three jojoba closely related species based on a functional annotation analysis in OmicsBox v2.0 for coding sequences (CDSs) from the jojoba male HiFi assembly. A close relationship was shown between jojoba and the following species: beetroot (Beta vulgaris subsp. vulgaris), quinoa (Chenopodium quinoa), and spinach (Spinacia oleracea). Figure S1. The k‐mer distribution and coverage of sequencing reads at K = 27 for (a) male and (b) female. Peaks with single and double asterisks were evaluated as k‐mer species derived from heterozygous (k‐mer frequency = 13) and homozygous (k‐mer frequency = 63) sequences for male and heterozygous (k‐mer frequency = 12) and homozygous (k‐mer frequency = 65) sequences for female. Figure S2. Syntenic comparison of the jojoba genome. A dot plot showing the syntenic gene pairs (totally 346) in the jojoba genome. Each dot shows one synteny gene pair (left: colour‐coded based on Ks rates in Figure 2). The grey lines (on the x and y‐axes) represent chromosomes. Figure S3. Ks analysis (synonymous d [file TPJ-108-1283-s001.docx]

**Supplementary material for online publication only**

**Sequencing of the jojoba genome reveals wide divergence of the sex chromosome in a dioecious plant**

Table S1: Jojoba male and female genome assemblies using Improved Phase Assembly (IPA) v:1.3.1

| Sex | Male | Female |
| --- | --- | --- |
| Total Length (bases) | 831,514,353 | 821,938,190 |
| Average Length (bases) | 3,149,675 | 2,634,417 |
| N50 length (bases) | 5,688,777 | 4,890,676 |
| L50 length (bases) | 49 | 59 |
| Longest (bases) | 21,809,893 | 13,633,347 |
| GC% | 37.31 | 37.31 |
| Contig No. | 264 | 312 |
| Length ≥ 1Mb | 264 | 312 |

Table S2: Quality assessment tool (QUAST) statistics of Jojoba (Simmondsia chinensis) male genome assembly using Dovetail HiRise v2.0.

| Statistic/Assembler | Dovetail HiRise |
| --- | --- |
| Total Length (bases) | 831,537,837 |
| Average Length (bases) | 20,281,410 |
| N50 length (bases) | 35,828,999 |
| L50 length (bases) | 11 |
| Longest (bases) | 49,657,746 |
| GC% | 37.31 |
| Contig No. | 41 |
| Length ≥ 1Mb | 41 |

Table S3: Jojoba 26 pseudochromosomes length based on chromosome-level dovetail HiRise assembly.

| # | Chromosome# | Length bp |
| --- | --- | --- |
| 1 | Chr1 | 49,657,746 |
| 2 | Chr2 | 42,968,296 |
| 3 | Chr3 | 42,177,036 |
| 4 | Chr4 | 40,273,687 |
| 5 | Chr5 | 40,184,469 |
| 6 | Chr6 | 39,723,960 |
| 7 | Chr7 | 39,324,133 |
| 8 | Chr8 | 38,596,718 |
| 9 | Chr9 | 37,647,928 |
| 10 | Chr10 | 37,281,067 |
| 11 | Chr11 | 35,828,999 |
| 12 | Chr12 | 35,108,529 |
| 13 | Chr13 | 34,930,154 |
| 14 | Chr14 | 33,843,129 |
| 15 | Chr15 | 31,357,304 |
| 16 | Chr16 | 31,020,504 |
| 17 | Chr17 | 26,988,044 |
| 18 | Chr18 | 26,093,974 |
| 19 | Chr19 | 25,223,309 |
| 20 | Chr20 | 24,577,473 |
| 21 | Chr21 | 23,935,793 |
| 22 | Chr22 | 20,026,881 |
| 23 | Chr23 | 19,485,935 |
| 24 | Chr24 | 18,259,458 |
| 25 | Chr25 | 17,909,950 |
| 26 | Chr26 | 17,424,492 |

Table S4: Benchmarking universal single-copy orthologs (BUSCO) analysis of Jojoba male results for both assemblers HiRise and Improved Phase Assembly (IPA).

| Assembler | Sex | Completed Genes | Single Copy | Duplicated | Fragmented | Total BUSCO Groups |
| --- | --- | --- | --- | --- | --- | --- |
| IPA | Male | 96.9% | 94.8% | 2.1% | 1.6% | 425 |
| HiRise | Male | 96.7% | 96.0% | 0.7% | 1.9% | 425 |

Table S5: The percentage of repeat family sequences in male and female Jojoba genomes v1.0.11.

| Repeat Family | Percentage of sequence% | |
| --- | --- | --- |
|  | Male | Female |
| LINESs | 4.94 | 4.97 |
| LTR elements | 32.44 | 32.01 |
| DNA elements | 3.65 | 3.25 |
| Unclassified | 29.00 | 28.27 |
| Simple repeats | 0.94 | 1.70 |
| Interspersed repeats | 70.03 | 68.50 |
| Total | 70.63 | 69.81 |

Table S6: The top four jojoba closely related species based on a functional annotation analysis in OmicsBox v2.0 for jojoba isoseq reference shows a close relationship between jojoba and the following species Quinoa (Chenopodium quinoa), Beetroot (Beta vulgaris subsp. vulgaris), Spinach (Spinacia oleracea) and Grape vine (Vitis vinifera) .

| # | Species | Order | Blast Hits# |
| --- | --- | --- | --- |
| 1 | Quinoa (Chenopodium quinoa) | [Caryophyllales](https://www.google.com/search?client=safari&sa=X&hl=en&sxsrf=ALeKk01B3GderoN5j1LqERm0VA-5yEDZ3g:1601333753418&q=Caryophyllales&stick=H4sIAAAAAAAAAONgVuLQz9U3MCysLFjEyuecWFSZX5BRmZOTmJNaDAC3L-oeHQAAAA&ved=2ahUKEwjG5KeM-YzsAhWC7XMBHTbCCeUQmxMoATB5egQIFBAD) | 431,080 |
| 2 | Beetroot ([Beta vulgaris subsp. vulgaris](https://en.wikipedia.org/w/index.php?title=Beta_vulgaris_subsp._vulgaris&action=edit&redlink=1)) | [Caryophyllales](https://www.google.com/search?client=safari&sa=X&hl=en&sxsrf=ALeKk01B3GderoN5j1LqERm0VA-5yEDZ3g:1601333753418&q=Caryophyllales&stick=H4sIAAAAAAAAAONgVuLQz9U3MCysLFjEyuecWFSZX5BRmZOTmJNaDAC3L-oeHQAAAA&ved=2ahUKEwjG5KeM-YzsAhWC7XMBHTbCCeUQmxMoATB5egQIFBAD) | 290,342 |
| 3 | Spinach (Spinacia oleracea) | [Caryophyllales](https://www.google.com/search?client=safari&sa=X&hl=en&sxsrf=ALeKk01B3GderoN5j1LqERm0VA-5yEDZ3g:1601333753418&q=Caryophyllales&stick=H4sIAAAAAAAAAONgVuLQz9U3MCysLFjEyuecWFSZX5BRmZOTmJNaDAC3L-oeHQAAAA&ved=2ahUKEwjG5KeM-YzsAhWC7XMBHTbCCeUQmxMoATB5egQIFBAD) | 288,400 |
| 4 | Grape vine (Vitis vinifera) | Vitales | 243,117 |

Table S7: The top three jojoba closely related species based on a functional annotation analysis in OmicsBox v2.0 for coding sequences (CDS) jojoba male from HiFi assembly shows a close relationship between jojoba and the following species, Beetroot (*Beta vulgaris* subsp. *vulgaris*), Quinoa (*Chenopodium quinoa*) and Spinach (*Spinacia oleracea*).

| # | Species | Order | Blast Hits# |
| --- | --- | --- | --- |
| 1 | Beetroot ([Beta vulgaris subsp. vulgaris](https://en.wikipedia.org/w/index.php?title=Beta_vulgaris_subsp._vulgaris&action=edit&redlink=1)) | [Caryophyllales](https://www.google.com/search?client=safari&sa=X&hl=en&sxsrf=ALeKk01B3GderoN5j1LqERm0VA-5yEDZ3g:1601333753418&q=Caryophyllales&stick=H4sIAAAAAAAAAONgVuLQz9U3MCysLFjEyuecWFSZX5BRmZOTmJNaDAC3L-oeHQAAAA&ved=2ahUKEwjG5KeM-YzsAhWC7XMBHTbCCeUQmxMoATB5egQIFBAD) | 4430 |
| 2 | Quinoa (Chenopodium quinoa) | [Caryophyllales](https://www.google.com/search?client=safari&sa=X&hl=en&sxsrf=ALeKk01B3GderoN5j1LqERm0VA-5yEDZ3g:1601333753418&q=Caryophyllales&stick=H4sIAAAAAAAAAONgVuLQz9U3MCysLFjEyuecWFSZX5BRmZOTmJNaDAC3L-oeHQAAAA&ved=2ahUKEwjG5KeM-YzsAhWC7XMBHTbCCeUQmxMoATB5egQIFBAD) | 3987 |
| 3 | Spinach (Spinacia oleracea) | [Caryophyllales](https://www.google.com/search?client=safari&sa=X&hl=en&sxsrf=ALeKk01B3GderoN5j1LqERm0VA-5yEDZ3g:1601333753418&q=Caryophyllales&stick=H4sIAAAAAAAAAONgVuLQz9U3MCysLFjEyuecWFSZX5BRmZOTmJNaDAC3L-oeHQAAAA&ved=2ahUKEwjG5KeM-YzsAhWC7XMBHTbCCeUQmxMoATB5egQIFBAD) | 2464 |

**Fig S1:** The k-mer distribution and coverage of sequencing reads at K = 27 for male (figure#a) and female (figure#b). Peaks with single and double asterisks were evaluated as k-mer species derived from heterozygous (k-mer frequency = 13) and homozygous (k-mer frequency = 63) sequences for male, and heterozygous (k-mer frequency = 12) and homozygous (k-mer frequency = 65) sequences for female.

**Whole genome duplication and lineage divergence in Jojoba**

The SynMap2 package was used for Ka analysis (Haug-Baltzell, 2017). The chromosome level genome assembly of jojoba along with its annotated file (gff3) including the gene details was uploaded into CoGe platform (Lyons and Freeling, 2008). The analysis was carried out using SynMap2 (Haug-Baltzell, 2017). DAGChainer parameter to detect chains of syntenic genes was set to "Relative Gene Order" with maximum 20 genes of distance between two matches and a minimum of 5 genes. The syntenic blocks were merged with “Quota Align” parameter. The in-built CodeML was used to calculate Ks substitution rate. The colour set sensitivity was set to 6.

Analysis of the syntenic regions in the 26 chromosomes of the jojoba genome identified 346 putative homologous genes using SynMap2 (Fig S2) (Haug-Baltzell, 2017). The Ks analysis also showed two whole genome duplication events. The mean value of largest Ks peak was _~_0.2, representing an ancient whole genome duplication (orange) (Fig S3). This was followed by a second (younger) WGD (red) with Ks value of 0.5. Panchy et al. (2016) suggested that on average 65% of genes in plant genomes have faced duplications. This is consistent with the theory that paleopolyploidization events are prevalent in the evolution of plant lineage. It seems that the jojoba genome has gone through a diploidization process (Conant et a., 2014). This is consistent with previous reports indicating that this species behaves like a diploid during mitosis and meiosis in cytological analysis (Tobe et al., 1992).


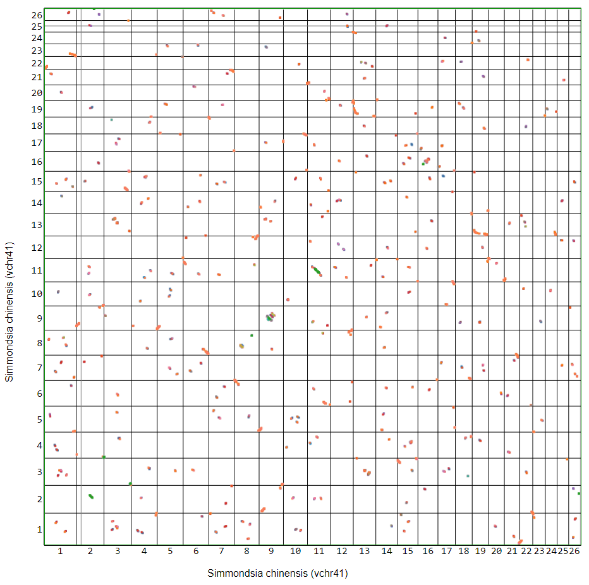


Fig S2. Syntenic comparison of jojoba genome. A dot-plot showing the syntenic gene pairs (totally 346) in jojoba genome. Each dot shows one synteny gene-pair (left: colour-coded based on Ks rates in Fig 2). The gray lines (in X and Y axis) represent chromosomes.


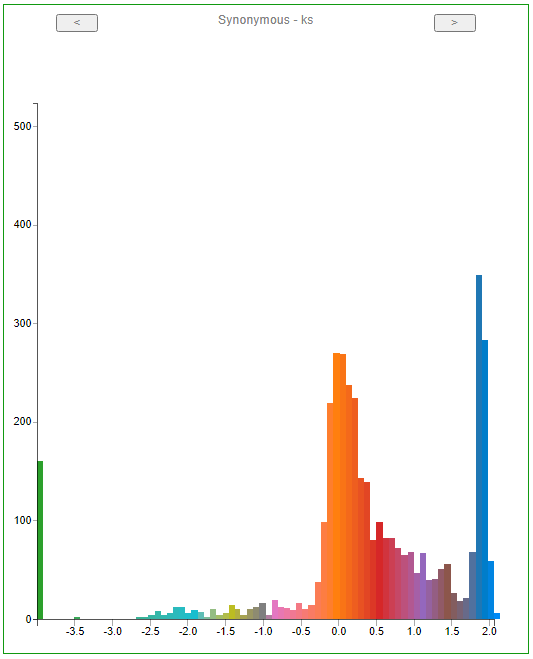


Fig S3. Ks analysis (synonymous distribution) of jojoba genome. The median peak (orange) with Ks value of 0.2 shows and early WGD event (Syntenic orthologs). The red peak with Ks value of 0.5 shows the younger (second) WGD (Syntenic out-paralogs). The green and blue columns are noises.

References

[Asher Haug-Baltzell, Sean A. Stephens, Sean Davey, Carlos E. Scheidegger, Eric Lyons; SynMap2 and SynMap3D: web-based whole-genome synteny browsers, Bioinformatics, Volume 33, Issue 14, 15 July 2017, Pages 2197–2198, https://doi.org/10.1093/bioinformatics/btx144](https://uq-my.sharepoint.com/personal/uqrhenr2_uq_edu_au/Documents/Desktop/Asher%20Haug-Baltzell,%20Sean%20A.%20Stephens,%20Sean%20Davey,%20Carlos%20E.%20Scheidegger,%20Eric%20Lyons;%20SynMap2%20and%20SynMap3D:%20web-based%20whole-genome%20synteny%20browsers,%20Bioinformatics,%20Volume%2033,%20Issue%2014,%2015%20July%202017,%20Pages%202197–2198,%20https:/doi.org/10.1093/bioinformatics/btx144)

Conant G. C., Birchler J. A., Pires J. C.. Dosage, duplication, and diploidization: Clarifying the interplay of multiple models for duplicate gene evolution over time. *Curr. Opin. Plant Biol.* 19, 91–98 (2014).

[Eric Lyons, Michael Freeling (2008) How to usefully compare homologous plant genes and chromosomes as DNA sequences The Plant Journal 53 (4) , 661-673](http://onlinelibrary.wiley.com/doi/10.1111/j.1365-313X.2007.03326.x/full)

Panchy et al: https://www.ncbi.nlm.nih.gov/pmc/articles/PMC4972278/
